# Influence of applying VARK learning styles on enhancing teaching skills: application of learning theories

**DOI:** 10.1186/s12909-024-05979-x

**Published:** 2024-09-26

**Authors:** Enas El-Saftawy, Ahmed A. Abdel Latif, Asmaa M. ShamsEldeen, Mansour A. Alghamdi, Amal M. Mahfoz, Basma Emad Aboulhoda

**Affiliations:** 1 Department of Medical Parasitology, Faculty of Medicine, Cairo University, Cairo, Egypt; 2 Department of Medical Parasitology, Armed Forces College of Medicine, Cairo, Egypt; 3 Department of Medical Parasitology, October 6th University, Healthcare Quality Excellence Diploma, Cairo, Egypt; 4 Department of Physiology, Faculty of Medicine, Cairo University, Giza, 11562 Egypt; 5 Department of Anatomy, College of Medicine, King Khalid University, Abha, 62529 Saudi Arabia; 6 Department of Pharmacology and Toxicology, Faculty of Pharmacy, Modern University for Technology and Information, Cairo, Egypt; 7 Department of Anatomy and Embryology, Faculty of Medicine, Cairo University, Cairo, Egypt

**Keywords:** Facebook, WhatsApp, VARK model, Zeigarnik’s effect, Memory storage, Retrieval strength

## Abstract

**Background:**

Social media in our networks have been exploited as dynamic learning tools and free platforms.

**Aims:**

The main objective of this study is to determine the impact of VARK learning styles (visual (V), aural (A), read/write (R), and kinesthetic (K)) in enhancing parasitological laboratory skills using social media and various learning theories.

**Methods:**

A research sample of 100 chemists working in Mega Alfa labs underwent online learning of laboratory parasitology skills via Facebook posts and WhatsApp dictated messages for an average of 7 weeks. All posts served various VARK learning styles and were designed based on Zeigarnik’s effect (conducting information with tactical breaks), memory storage and retrieval strength theories (repetition of information). Trainees were classified according to their VARK learning style preferences and were evaluated through pre/post-tests. Data on VARK learning styles were summarized using frequency (count) and relative frequency (percentage). Data of pre-test and post-test scores were summarized using mean and standard deviation. T-test was used to compare pre-test and post-test scores. The difference between the pre-test results, the post-test results and the preferred learning style was analyzed using ANOVA with Tukey’s post-hoc testing. P-values less than 0.05 were considered statistically significant.

**Results:**

In a total of 100 trainees, tri-modal and multimodal learning styles were preferred by 40% and 30% of the trainees respectively; on the contrary, the unimodal and bimodal learning styles were the least preferred. In the trimodal and multimodal groups, the post-test results showed significant increase when compared with the pre-test results. Also, using the ANOVA test and a Tukey’s post-hoc comparison, the assemblage of multiple learning styles (tri-modal and multimodal) appeared to significantly improve the learning performance in the post-test results when compared with the unimodal and bimodal groups.

**Conclusion:**

The tri-modal and multimodal learning styles were found to influence the acquirement of the laboratory parasitology skills much better than the unimodal and bimodal learning styles. Kinesthetic learning should have a special emphasis in training.

## Introduction

The representations of education may understate vital facets in the real life of working in a lab [1]. Sumin et al. (2018) deduced the importance of knowledge application to gain effective learning [2]. Parasitology has been regarded as a basic science in the faculty of science graduating chemists. Nevertheless, a gap is always present between the faculty theoretical education and so far, the laboratory practical application of knowledge. Of importance, Egypt is an endemic region for neglected tropical parasites due to its climatic and environmental conditions. Hence, it is necessary to be concerned with learning laboratory skills in parasitology to renovate human power into a productive and expert output, enhance the practical skills in diagnosis of infections, and improve public health [3].

Application of the different VARK variants including the visual (V), audio (A), read/write (R), and kinesthetic (K) learning styles is of special importance in the practical activities related to the laboratory parasitology skills. It is noteworthy that the laboratory work of parasitology determines a wide aspect of skills e.g., proper sampling acquisition, preparation and assessment of the biological samples (stool sample, urine sample, vaginal swab, Scotch adhesive tape swab), special stains, identification of helminthes and protozoa both macroscopically and microscopically, immunological tests, molecular techniques for parasitic infections and detection of the diagnostic stages of the parasites (depending on definitive criteria of the organisms, pattern and nature of the parasite motility (jerky movement, progressive movement) which all necessitate combination of the visual (V), auditory (A), (R) read/write and kinesthetic (K) learning styles [4]. Innovating those training skills into brain-based VARK learning styles enhances learning achievement by understanding memory and inspiration for practical application of the theoretical knowledge [5].

The VARK scale is one of the most famous learning style models that was introduced by Neil Fleming in 2006 and was assessed by the University of Florida. Previous inventories of models for the VARK brain-based learning styles revealed that the candidates showed variations in their intellectual styles [6–9]. The visual style almost manipulates arrows, drawings, and models, that symbolize printed information. Audi learners (A) deliver attention to lyrics brought by their teachers and mp3 recordings to get detailed notes, read/write learners (R) keepsake to the printed detailed lectures and textbooks to obtain information, and kinesthetic learners (K) prefer practical sessions and application to obtain real-life experience [10].

Several previous studies have explored the learning styles and performance of trainees. For example, Tonkaboni et al. [9] demonstrated that students with increased educational levels are more inclined to manipulate the kinetic style of learning rather than the read/write learning modal. Khanal et al. (2019) [11] speculated that undergraduate students are different in their preferred learning styles, yet, most students are multimodal. Similar results were declared by Inam and Haq (2022) [12] who conducted their study on final-year MBBS students.

The Russian Psychologist Dr. Bluma Zeigarnik introduced the theory known as the Zeigarnik effect to enhance knowledge acquisition [13]. The theory deduces that people tend to memorize incomplete missions better than finished ones. When a job remains incomplete, it generates a kind of pressure, annoyance, suspense, suspicion, and concern about the consequences; therefore, it becomes hard to forget. In this context, interruption of the learning process has been exhibited as a valuable learning policy and a good technique for increasing the conception and preservation of information. Jean, 2019 [14] stated that Zeigarnik’s effect attracts psychological attention to the real goals.

Besides, the theory of memory storage and retrieval recovery relies on the repetition and reinforcement of information to guarantee profound storage. Such a strategy emphasizes thinking and application of information especially in learning new vocabulary, metaphors, themes, and Fig. [15].

Social media enable people to get close, find career opportunities, and connect with communities across the earth with similar interests. There are different applications that serve social media. For example, Facebook and WhatsApp. Interestingly, social media have been considered a training platform. However, the success of conducting a training program is related to the ability of the trainers to engage trainees in the program content, achieve proficient organization, and make clear intended learning objectives that are closely-related to practice improvement [16–19].

This study aims to gain a deeper understanding of how online virtual social media platforms such as WhatsApp and Facebook can be incorporated into different learning styles (VARK) in a way enhanced by different learning theories (Zeigarnik effect, memory storage and retrieval recovery). Moreover, the preferences and effects of the different learning styles among trainees were assessed through pre/post-test.

Our study is the first, to the best of our knowledge, to investigate the learning style preferences on the chemist trainees, while examining the effects of the learning style preference on the proficiency of the lab skills in the context of the differences in the pre/post-test results. While previous studies have investigated the VARK learning style preference in medicine [20], pharmacy [21], engineering [22], nursing [23], and dental specialties [24], yet, no previous study has examined the real-life implication of this learning style preference on the proficiency of the laboratory skills.

## Methodology

### Study setting

The current study was carried out with the cooperation of the training units related to the Mega Alfa labs starting from October 2018 to December 2021.

### Study population

A cross-sectional study was conducted on a total of 100 chemists, 61 were male and 39 females, aged between 22 years to 25 years old. A pre-post design study was conducted. Thirteen OSPE stations were constructed to simulate multiple tasks and to assess certain intended learning objectives (ILOs) [25]. Inclusion criteria: recently graduated chemists who are receiving training to be hired. Exclusion criteria: chemists who are already hired in the workplace and graduated for more than 1 year. The sample size is calculated using the following formula: *n* = 2 (Za + Z1–β) 2σ2, Δ2 where *n* = 100 [26].

### Ethical consideration

The aim of the study was described to the trainees, and written consent was obtained before conducting the VARK questionnaire. Chemists were free to accept or refuse the filling of the questionnaire by the end of the training course. Also, they were free not to continue training at any time. The study was approved by the Institutional Review Board and Ethical Committee of 6th October University (approval number PRC-Me-2311008). Before enrolment, written informed consent was obtained from all the participants, wherein they were informed that they could freely withdraw from the study. There would be no negative consequences from opting not to participate in the study. All methods were carried out in accordance with relevant guidelines and regulations, including the Code of Practice for the Care and Use of Human Subjects for Scientific Purposes according to the Declaration of Helsinki (2013).

### Pretest and post-test assessment

Pre-tests and post-tests were conducted at the start and end of the three training courses consecutively to test the acquisition of the trainees to the training course’s intended learning objectives (ILOs). Each test was composed of thirteen questions (3 case studies, 4 MCQ questions, and 6 microscopic identifications), and each question scored one mark, i.e. 13 is the maximum score.

### The learning material

The current study introduced *medical contexts for parasitic diseases*, various laboratory skills “safety, quality, …”, labeled mini-videos (40 ± 10 s.) for microscopic fields supplied with narrated commentary, and discussions on the most commonly asked questions. The material was subdivided into three consecutive courses.

### The online platform

- A WhatsApp group Data were executed in the form of dictated messages. Each course was for consecutive 50 ± 5 days (i.e. average of 7 weeks).
- A Facebook page Exploiting the habit of scrolling through daily posts a Facebook page was constructed with the hyperlink https://www.facebook.com/Parasitology-for-All-112721744195736.

The authors have chosen social media as an e-learning platform to deliver the training material in visual, auditory and kinesthetic modalities for a variety of reasons including the modern behavioral patterns in frequently navigating to the social media, preference of network technology for transfer of information and interest of the trainees in the online teaching approach rather than the traditional education. The social media is also cost-effective being a feasible tool in the hands of every participant. The social media platforms also offered the trainees schedule flexibility, easy interaction and simple achievement orientation. Investigating how the trainees learning styles affect the quality of post-test performance is also better achieved through the social media. The social media also represented a suitable platform for delivering the different VARK learning style variables that engage the participants’ kinesthetic, auditory, visual, and read-write senses through different multimedia forms (Videos, text, photographs, audios, graphics and interactive material etc.). This endeavor would yield a more accurate illustration of the learning style preferences of the laboratory trainees and the effect of incorporation of these learning styles on the real-life practical proficiency in the lab field.

### Core features of the training courses

**Fig. 1:**
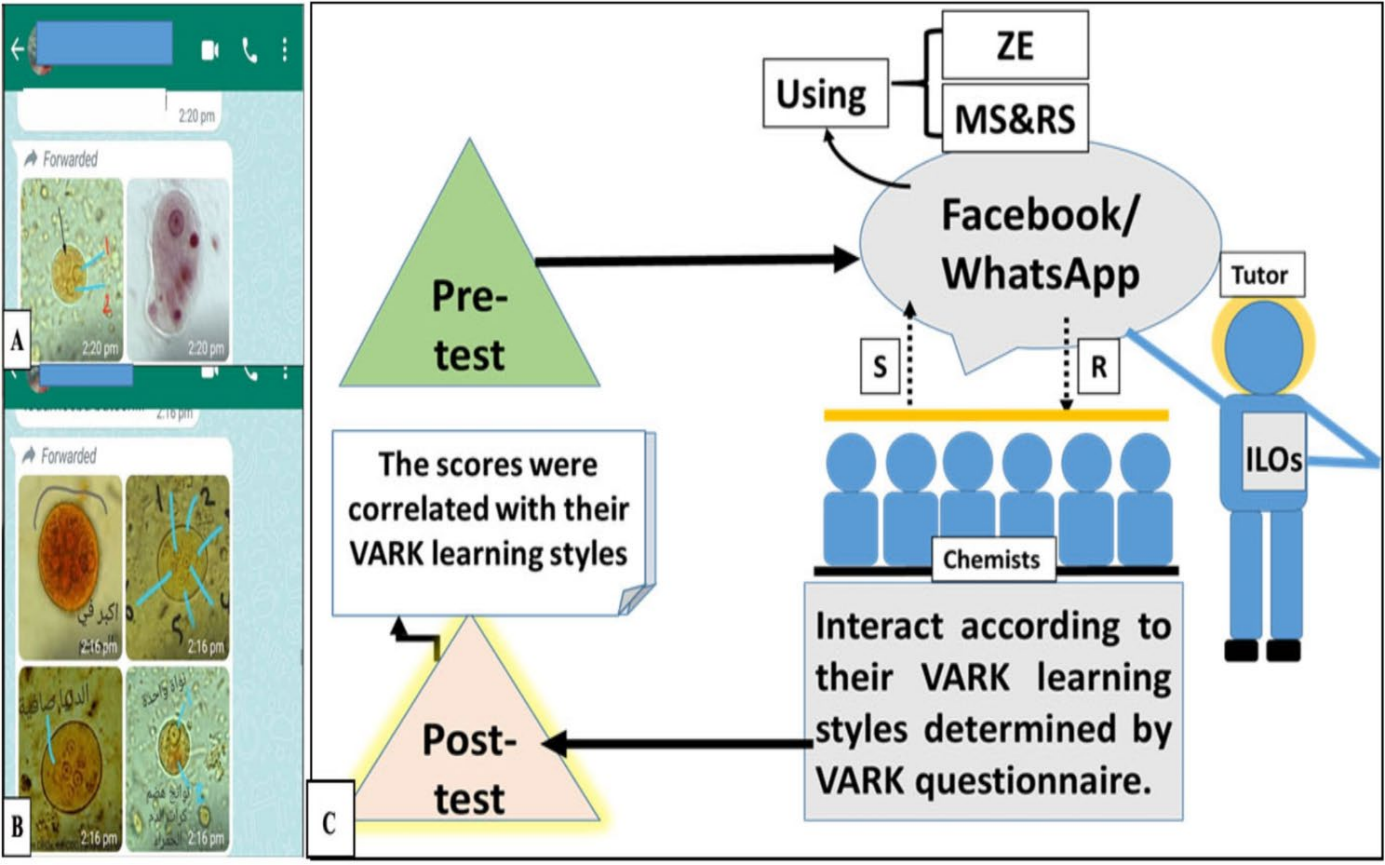
Conduction of educational material. **(A)**, **(B)** A model for the dictated data in the WhatsApp group with labelled figures for different parasites. **(C)** The whole procedure of the online training. ZE: Zeigarnik’s effect; MS&RS: memory storage and retrieval strengths theory; R: receive posts; S: send anonymous microscopic structures and inquiries

- Scripts Scripts were manipulated to distribute the information among chemists irrespective of their levels of expertise. Overall, the author used a simple display of information to encourage engagement and interactivity [27, 28].
- The applied theories The educational material was published and shared regularly, repeatedly, and in pieces applying the theory of memory storage and retrieval recovery strengths and Zeigarnik’s effect; additionally, all material was designed to serve two or more components of the VARK learning styles. **Visual** in the form of labeled images in mini-videos and posts; **auditory** in the form of audio commentary in the mini-videos and explanations; **reading/writing** in the form of textual reviews and discussions; and **kinesthetic** through encouraging the trainees for the digital sharing of images and videos captured during their real work life. Posts involved figures that were labeled by the author as in Fig. 1. Chemists were grouped according to their VARK preferences and underwent post-test assessment by the end of the training course.
- Repetitive publication of the videos and photos was done to increase interaction and involvement by the trainees through their comments. The trainees were provoked to ask the trainer on the WhatsApp group to accomplish the previous incompletely explained slide to achieve the information and discriminate different parasites microscopically; thus, attaining interaction by the trainees using the Zeigarnik effect and memory storage and retrieval recovery theories.

### Assessment of VARK style

**Fig. 2:**
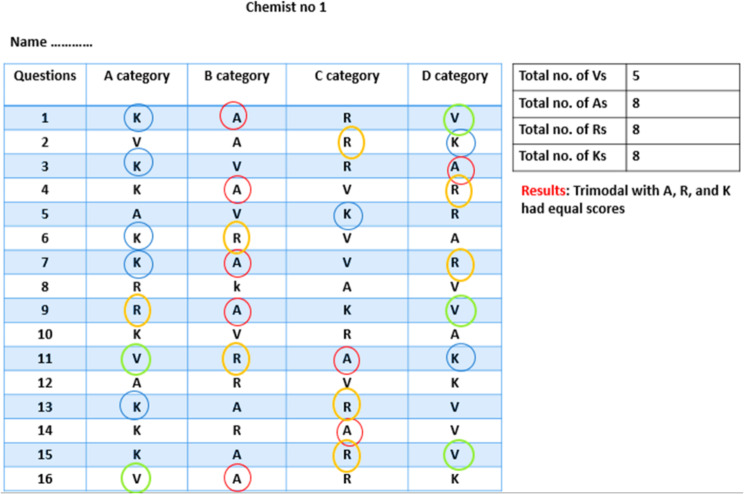
VARK questionnaire and the manner of interpretation

1. A printed form of the VARK questionnaire was manipulated that entailed 16 MCQ questions with 4 choices for each and was distributed to the participants at the end of the training course. Each choice relates to the preference for a precise sensory modality. Therefore, the modality that got the maximum marks was the favorite sensory style. Since trainees were allowed to choose more than one option, multiple styles of varying patterns could be achieved. In the case of two, three, or four learning styles scoring equal records, bi-, tri-, or multi-modal learning styles are determined, respectively. The questionnaire followed formerly validated scoring guidelines [10] (Fig. 2).

### Statistical analysis and operational definitions

Data were coded and entered using the statistical package for the Social Sciences (SPSS) version 28 (IBM Corp., Armonk, NY, USA). Data on VARK learning styles were summarized using frequency (count) and relative frequency (percentage). Data of pre-test and post-test scores were summarized using mean and standard deviation. The T-test was used to compare pre-test and post-test scores. The difference between the pre-test results, the post-test results and the preferred learning style was analyzed using ANOVA with Tukey’s post-hoc testing. P-values less than 0.05 were considered statistically significant.

## Results

Sixty-two videos for laboratory skills and microscopic identification, a collection stock of 254 high-resolution parasite photos, and 64 reviews were conducted by the trainer and trainees using their work equipment.

### Descriptive analysis of the preferences of the VARK learning styles

**Fig. 3:**
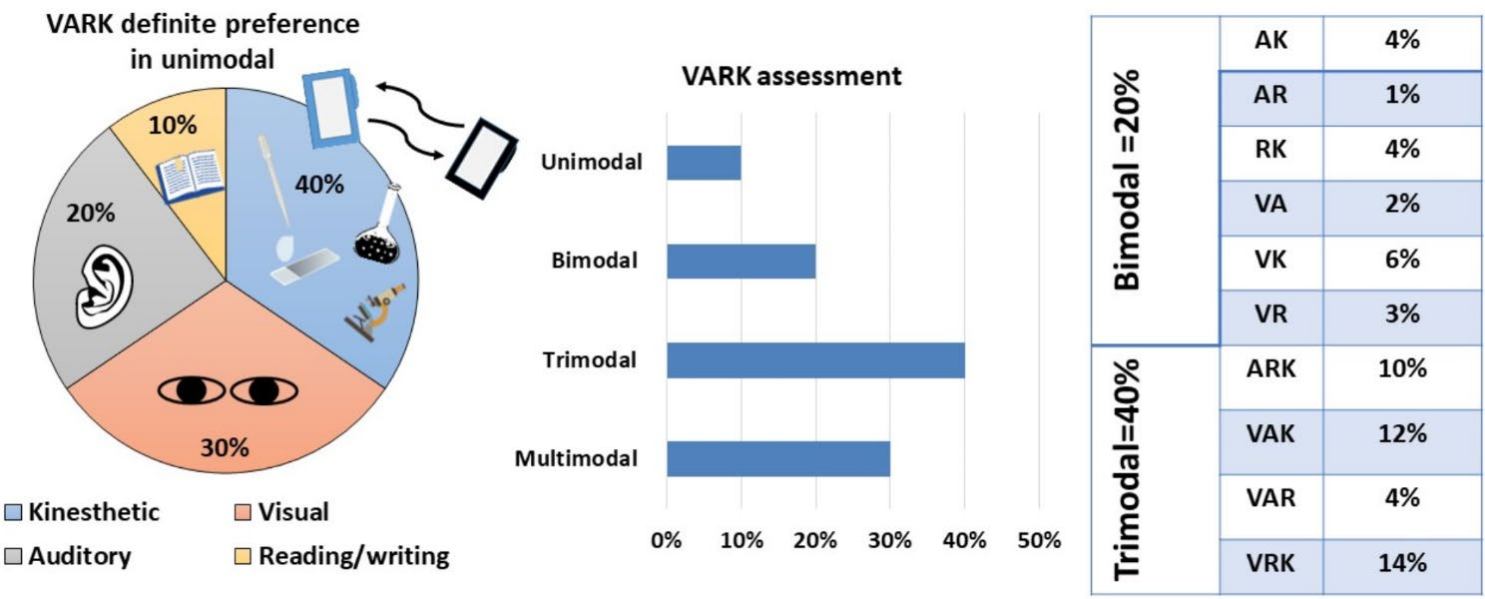
VARK assessment. **A** Distribution of VARK within the unimodal (single preference). **B** and ***C*** Distribution of the bi, tri, and multimodal learning styles

Results of the trainees’ questionnaire revealed that out of 100 chemists, the tri-modal style of learning was predominantly preferred (*n* = 40, 40% of the trainees) followed by the multimodal style of learning (*n* = 30, 30% of the trainees). Notably, in the trimodal style of learning, kinesthetic (active) learning provided with other learning styles had the highest records. Nevertheless, the unimodal and bimodal styles had lower scores (10% and 20%, respectively). The results also revealed that a definite single preference was noticed in only 10% of the chemists, where the predominantly preferred learning style was the kinesthetic followed by the visual style (Fig. 3).

### Pre- and post-scores in the different groups of the VARK learning styles

The mean values of post-test results were (1.530 ± 0.376, 1.530 ± 0.278, 12.278 ± 0.184, and 12.299 ± 0.215) in the unimodal, bimodal, trimodal, and multi-modal styles, respectively. Using the T-test, the pre-test and post-test scores in both the trimodal and multimodal groups revealed significant differences (Table 1).

**Table 1:** Pre- and post-scores in the different groups of the VARK learning styles

	Unimodal		Bimodal		Trimodal		Multimodal	
	Mean	Standard deviation	Mean	Standard deviation	Mean	Standard deviation	Mean	Standard deviation
**Pre-test scores**	1.30	0.82	1.30	0.80	2.65	1.00	2.80	1.10
**Post-test scores**	1.40	0.52	1.40	0.50	12.33	1.25	12.37	1.38
**P-value**	0.678		0.541		< 0.001		< 0.001	

### Post-test results in the different groups of the VARK learning styles

Comparison between the effect of the VARK learning style on the post-test results using ANOVA test with Tukey’s post-hoc test showed that the tri-modal and multimodal learning styles had better performance with statistically significant difference (p-value < 0.001) when compared with the unimodal and bimodal learning styles as shown in Table 2.

**Table 2:** Post-test assessment using the hoc pairwise comparison test between groups

Group		*P* value
**Unimodal learning style**	**Bimodal learning style**	1.000
	**Trimodal learning style**	< 0.001
	**Multimodal learning style**	< 0.001
**Bimodal learning style**	**Unimodal learning style**	1.000
	**Trimodal learning style**	< 0.001
	**Multimodal learning style**	< 0.001
**Trimodal learning style**	**Unimodal learning style**	< 0.001
	**Bimodal learning style**	< 0.001
	**Multimodal learning style**	0.936
**Multimodal learning style**	**Unimodal learning style**	< 0.001
	**Bimodal learning style**	< 0.001
	**Trimodal learning style**	0.936

### Based on estimated marginal means

## Discussion

The current study has been designed to investigate the learning style preferences of the chemist trainees, while examining the effects of those learning styles on the proficiency of the lab skills in the context of the differences of the pre/post-test results.

Knowledge about learning styles is crucial for the progress of medical education, especially in the health-related disciplines [29]. In the current study, about 70% of the trainees showed a trimodal and multimodal learning preference, which is consistent with the previous learning style preference recorded on investigations of medical [20] and pharmacy [21] students denoting the role of multimodal learning on enhancing brain perception and achievement of learning goals [30]. Our results are also in agreement with the results of a questionnaire-based study supporting the effects of VARK-dependent learning in physician assistant [31] and management programs [32]. Ghobain and Zughaibi [33] speculated that multimodal education is a considerable chance for better education. Previous neuroscience research has also revealed that multiple representations of content using multimedia improved learning outcomes across the different learning styles [34]. Taken together, those results highlight the necessity of integrating the different learning styles to fit the multiple training needs.

The current model showed that the kinesthetic style was almost preferred either solely (unimodal) or in combination with other learning styles (bimodal, trimodal, and multimodal) followed by the visual style. This was reflected by improved performance of the trainees in the techniques of biological sample preparation and identification of pathogens. A plausible explanation to the kinesthetic preference among the participants is the practical nature of the laboratory field and the importance of accurate manipulation of the techniques in the trainees’ professional development. The trainees endorsed increased interest and reliance on shifting to practical and laboratory training rather than the academic didactic-focused teaching in the faculty of science graduating chemists. This preference also highlights the critical need for incorporating in-depth practical training for the newly-graduated chemist candidates before receiving an occupation in a diagnostic laboratory. This helps in interpretation of the assimilated theoretical knowledge into real-life laboratory skills that serve the future career.

Previous studies have reported variable learning style preferences in different studied fields. For example in the field of nursing, visual learning was the most preferred style, followed by the kinesthetic and auditory styles [35]. Also, dental students prefered visual learning to the kinesthetic style [36]. A plausible explanation of the discrepancy between our results and the aforementioned is that the learning style preference appears to be tailored according to the needs of the trainees to proficiently fill their deficiencies. It also differs according to the field of study and the ILOs required from the training.

Assemblage of multiple learning styles (tri-modal and multimodal) appeared to significantly improve the post-test results when compared with the unimodal and bimodal groups. This result reflects the advantageous effect of training using various learning styles to enhance knowledge recognition and practical proficiencies which gives the multimodal learning trainees an advantage over other peers.

Integration of the sensory and motor inputs appeared to affect the perception and processing of information, thereby helping in the practical training process. Comparable results have been reported by Tabatabei (2018) [31] who confirmed the crucial role of manipulating various senses with the VARK learning styles in enhancing deep learning. Similarly, Liu (2021) [37] has highlighted the importance of implementing multiple sensory inputs in the success of the training process. Dantas and Cunha (2020) [38] speculated that a successful learning process achieved through integration of different learning styles leads to neuronal interconnections in the cerebral cortex. This might reflect the provoking effects of the learning styles on the interpersonal attitude toward sharing and searching for knowledge, internal motivation, behavior, and practice [39].

According to Felder [40] each learning style has its potencies and flaws if manipulated individually. For example, addressing distant blended multimodal learning styles requires good online learning experiences [41], but it has, on the other hand, immense promises for improving the efficiency of distance learning and empowering deeper understanding of the subject matter [31, 36].

Recent studies have also illustrated the crucial role of enhancing the learning approaches using modern methods such as the tree machine learning model for optimizing the practice in the dental field [42], the mixed reality technologies to enhance in the surgical skills [43] and the educational video resources to improve teaching of clinical examination [44].

## Conclusion

This study is the first to illustrate the learning style preferences of chemist trainees and examine how such preferences affect lab skill competency in the light of the variations in pre- and post-OSCE test outcomes.

The study represents a promising training model for bridging the gap between the traditional theoretical faculty education and the hands-on laboratory skills required for practicing applied parasitology through implementation of the different components of the VARK learning styles via investing in the technology in hands, (i.e. cell phones and social media applications).

Since different individuals have unique preferences for learning, thus understanding and applying the visual, auditory, reading/writing, and kinesthetic models is a key approach for fostering an inclusive learning style based on each individual preference. The study, thereby, has tailored the practical material of the parasitology training to fit the various VARK learning styles. The Zeigarnik approach (implemented through short videos and petite didactic notes) and the retrieval recovery approach (applied through repeated material publication) appeared to enhance the model’s results and the memory storage of knowledge. This was reflected on the trainees’ preference of the tri-modal and multimodal strategies of the VARK model. The trimodal and multimodal approaches were also related to the highest post-test achievements among the trainees; which again elucidates the beneficial role of exposure to multiple sensory and motor inputs.

While the different VARK learning strategies now represent an area of extensive research in the practical medical, paramedical and pharmaceutical fields, yet, application of the VARK educational design for the purpose of mastering the laboratory skills, could pave the way to best personalize teaching not only to comprehend and retain information, but also to develop an engaging and efficient learning experience in a deeper, faster, and more comprehensive way, which, in turn, leads to optimal practical performance, enhanced motivation and improved employee engagement.

### Recommendations

Future large multicenter studies testing the implementation of the multimodal learning styles in the different practical laboratory sub-fields (microbiology, biochemistry, hematology, biotechnology, computational analysis, etc.) are recommended to build an encompassing framework for identifying the ideal educational design for accommodating the diverse learning preferences and develop the best ways of optimizing the required skills in a personalized learning approach according to the specific laboratory specialties.

## Data Availability

The data used and analyzed during the current study are available from the corresponding author upon reasonable request.
